# Computer-Tailored Student Support in Introductory Physics

**DOI:** 10.1371/journal.pone.0137001

**Published:** 2015-09-09

**Authors:** Madeline Huberth, Patricia Chen, Jared Tritz, Timothy A. McKay

**Affiliations:** 1 Center for Computer Research in Music and Acoustics, Department of Music, Stanford University, Stanford, CA, United States of America; 2 Department of Psychology, University of Michigan, Ann Arbor, MI, United States of America; 3 School of Information, University of Michigan, Ann Arbor, MI, United States of America; 4 Department of Physics, Department of Astronomy, and School of Education, University of Michigan, Ann Arbor, MI, United States of America; Universidad Veracruzana, MEXICO

## Abstract

Large introductory courses are at a disadvantage in providing personalized guidance and advice for students during the semester. We introduce E^2^Coach (an Expert Electronic Coaching system), which allows instructors to personalize their communication with thousands of students. We describe the E^2^Coach system, the nature of the personalized support it provides, and the features of the students who did (and did not) opt-in to using it during the first three terms of its use in four introductory physics courses at the University of Michigan. Defining a ‘better-than-expected’ measure of performance, we compare outcomes for students who used E^2^Coach to those who did not. We found that moderate and high E^2^Coach usage was associated with improved performance. This performance boost was prominent among high users, who improved by 0.18 letter grades on average when compared to nonusers with similar incoming GPAs. This improvement in performance was comparable across both genders. E^2^Coach represents one way to use technology to personalize education at scale, contributing to the move towards individualized learning that is becoming more attainable in the 21^st^ century.

## Introduction

Students in introductory physics courses are diverse. They arrive with a broad array of technical preparation, reasons for taking the courses, long-term career goals, affect toward and sense of identification with physics, personal and financial circumstances, and general study skills. However, most traditional forms of instruction for introductory courses treat students similarly. They have one set of lectures, homework, and exams, often tell students about the same applications, and provide the same advice. This format is partially motivated by necessity—especially at large institutions, many students take these courses. Indeed, at the University of Michigan (henceforth referred to as Michigan), about 1,100 students begin a two semester physics sequence every semester. Due to the scale of the courses and traditions developed to handle it, recognizing and tailoring to the diversity of the student population is often lost.

In an ideal world, we would provide an expert coach for each student; aware of their background, goals, and current status, experienced with success in physics, and able to deliver feedback, encouragement, and advice in the manner most likely to be effective. Such an informed, experienced coach would be well positioned to effectively encourage improved performance from each student. Since this is not currently practical, we have adapted a technology originally developed for personalized public health interventions [1]—computer tailored communication—as a substitute.

There are many reasons to expect that personalization will help students. Pedagogues in the fields of physics and engineering note the importance of teaching with different learning styles in mind [2, 3], and adaptive learning tools and environments are personalizing the learning process such that student needs can be immediately identified and content can be changed to better challenge the student [4]. Indeed, students arrive in introductory physics classes with a wide variety of technical preparation for study. At Michigan, students’ prior study of physics varies widely, ranging from none at all to two years of AP courses. Those who have not studied physics begin substantially behind their peers. Those who have often arrive with strong but inaccurate preconceptions about what success in physics requires. Math preparation is similarly diverse. Credit for college calculus is an advisory prerequisite for all of our introductory courses. Students meet this requirement either through AP calculus taken in high school or in one of a variety of first year calculus courses at Michigan or another college. While almost all students take introductory physics as a requirement for a course of study they hope to pursue, the degree to which they personally identify with physics varies widely. This degree of identification affects both student motivation and mindset, and may have a strong effect on their physics performance. By better understanding the state of each student, we have the opportunity to address each student in the most appropriate way, ideally in a manner which acknowledges their individuality.

Personalization of messages is important for a second, less obvious class of reasons. Public health research into computer tailoring has shown conclusively that messages tailored to a recipient’s identity are much more likely to affect behavior than those that are generic [5–8]. They have also found that impact is enhanced when participants read multiple tailored messages [9]. While intrinsic motivation is also an important factor in the outcome of the tailoring [10], overall, tailored messages have been effective in stimulating health behavior change with an effect size of slightly less than “small” magnitude [11]. Specifically with regards to teaching, not only does tailoring allow the message delivered to each student to be more appropriate, tailoring provides the opportunity to make the message more effective, particularly for students who are already intrinsically motivated.

This paper describes the application of a computer tailored communication system called E^2^Coach (an **E**xpert **E**lectronic **Coach**ing system) to more than a thousand students taking introductory physics courses at Michigan over three semesters. We begin with background and motivation for our attempt to personalize student support. This is followed by a brief description of the features of our E^2^Coach system as implemented in the winter 2012, fall 2012, and winter 2013 semesters, our analysis of the impact of E^2^Coach usage on student performance, and finally, a summary of our results, some lessons learned, and plans for the future. A table of abbreviations and acronyms used in this paper is available in Table 1.

**Table 1:** Acronyms used in this paper, listed alphabetically.

**Term**	**Definition**
ACT	American College Testing. A standardized test for high school achievement and college admissions.
AP	Advanced Placement. Exams taken by students typically while in high school that test specific subject areas.
BTE score	Better-Than-Expected score, a metric that describes a student’s physics performance with that of other students in the class with a similar GPA_other_.
GPA	Grade Point Average, a measure of performance in college classes. It is the average of all grades of all classes taken.
GPA_other_	GPA at the end of the term in which a student took a physics course *with the impact of the physics course itself removed*.
SAT	Scholastic Aptitude Test. A standardized test for high school achievement and college admissions.
SLC	Science Learning Center, a University of Michigan study resource for science classes, open to all students.
STEM	Science, Technology, Engineering and Mathematics.
Michigan	University of Michigan.

### Introductory Physics at Michigan

Introductory physics at Michigan is offered in three parallel two semester sequences. Physics 140 and 240 make up a two semester introduction to physics for those intending to major in the physical sciences or engineering, called simply *General Physics*. These courses are typically taken early in a student’s career, most often begun in the second semester of their first year on campus. Most students in this sequence come from Michigan’s College of Engineering (70–80%). Enrollment in this sequence shows the same substantial gender imbalance, with women making up only 27% of the class.

Physics 135 and 235 (formerly 125 and 126) comprise a two semester *Physics for the Life Sciences* sequence. These courses serve a diverse population of students either planning majors in life science disciplines or preparing themselves to pursue careers in the health sciences—approximately 75% plan to attend medical school after college. Students begin this sequence at many different points in their college careers, from their first semester on campus to their fifth year. These courses are more gender balanced, with women making up 56% of the class. Michigan also offers a third track, Physics 160 and 260; a small *Honors Physics* sequence that we do not discuss further here.

#### Quantifying student performance in physics classes

The genesis of this project lies with an effort to better understand the impact of student background and preparation on performance in physics, as measured by course grades. We acknowledge that the performance assessment provided by grades may be only loosely related to student learning. Nevertheless, grades play a central role in institutional assessment of student success and provide the principal feedback that students receive. As such, they play an important role in the student experience.

To explore the relation between background and student performance, we utilized student record data, extracting information about a group of 36,701 students who completed physics courses in the period from winter 1998 to winter 2008, a total of 31 semesters. Available information included a portrait of each student at the start of their physics class, some details of their performance in class, and a record of their final grade. Background information included high school GPA, standardized test scores (Michigan requires either students to take either the SAT or the ACT exam for consideration of admission), gender and other demographic information, and a summary of their performance in other courses at Michigan (GPA_other_). For this last, we calculated each student’s GPA at the end of the term in which that physics course was taken *with the impact of the physics course itself removed*. GPA_other_, while not available before the term, allows us to compare each student’s physics performance to their performance in other courses at Michigan, even when they take physics in their first term on campus.

As an initial test, we examined the relationship between physics final letter grades and standard college performance predictors—SAT score, ACT score, and GPA_other_, utilizing the subset of students who reported both SAT and ACT scores (N = 15,187). Correlations between these predictors and final grade are presented in Table 2. It is clear that GPA_other_ has the strongest correlation with physics letter grades. This strong relation between local GPA and course grades has been noted elsewhere [12]. It is hardly surprising that average grade in courses at Michigan effectively predicts grades in a course at Michigan. Unless the course of interest is utterly unlike other Michigan courses, this must be true.

**Table 2:** GPA_other_ has the strongest correlation with physics letter grades, when compared to SAT and ACT scores in physics students (1998–2008).

	**Final Grade**	**GPA_other_**	**SAT Score**	**ACT Score**
Final Grade	-	.66**	.35**	.33**
GPA_other_		-	.34**	.34**
SAT Score			-	.79**
ACT Score				-

Correlations *r*s are reported.

** indicates statistical significance at the *p* <.01 level.

To further examine the relative predictive power of standardized test scores and GPA_other_ on physics final letter grades, we conducted a hierarchical regression analysis using data from students with all three scores. SAT and ACT scores were entered in step 1 of the regression, and SAT, ACT, and GPA_other_ were entered in step 2. Table 3 presents the regression coefficients and the variance accounted for in the physics final grades (Δ*R* ^2^). As shown in step 2, GPA_other_ significantly predicted students’ final grade in physics even when controlling for SAT and ACT scores. GPA_other_ independently accounted for 32% of the variance in students’ final physics grades, which was statistically significant when controlling for the effects of standardized test scores. As shown in step 1, SAT and ACT scores predicted only 13% of the variance in student’s final physics grades when GPA_other_ was not included in the model. This analysis confirms that, of the three predictors, GPA_other_ bears the strongest relation to final grades in introductory physics classes.

**Table 3:** GPA_other_ significantly improves the prediction of physics final grades, above and beyond SAT Scores and ACT Scores, as shown in the hierarchical regression (N = 15,187).

**Variable**	***B***	***β***	**Δ*R*^2^**
**Step 1**			.13**
SAT Score	.00**	.25	
ACT Score	.04**	.13	
**Step 2**			.32**
SAT Score	.00**	.15	
ACT Score	.00	.01	
GPA_other_	1.02**	.61	

** indicates statistical significance at the *p* <.01 level.

**Fig 1:**
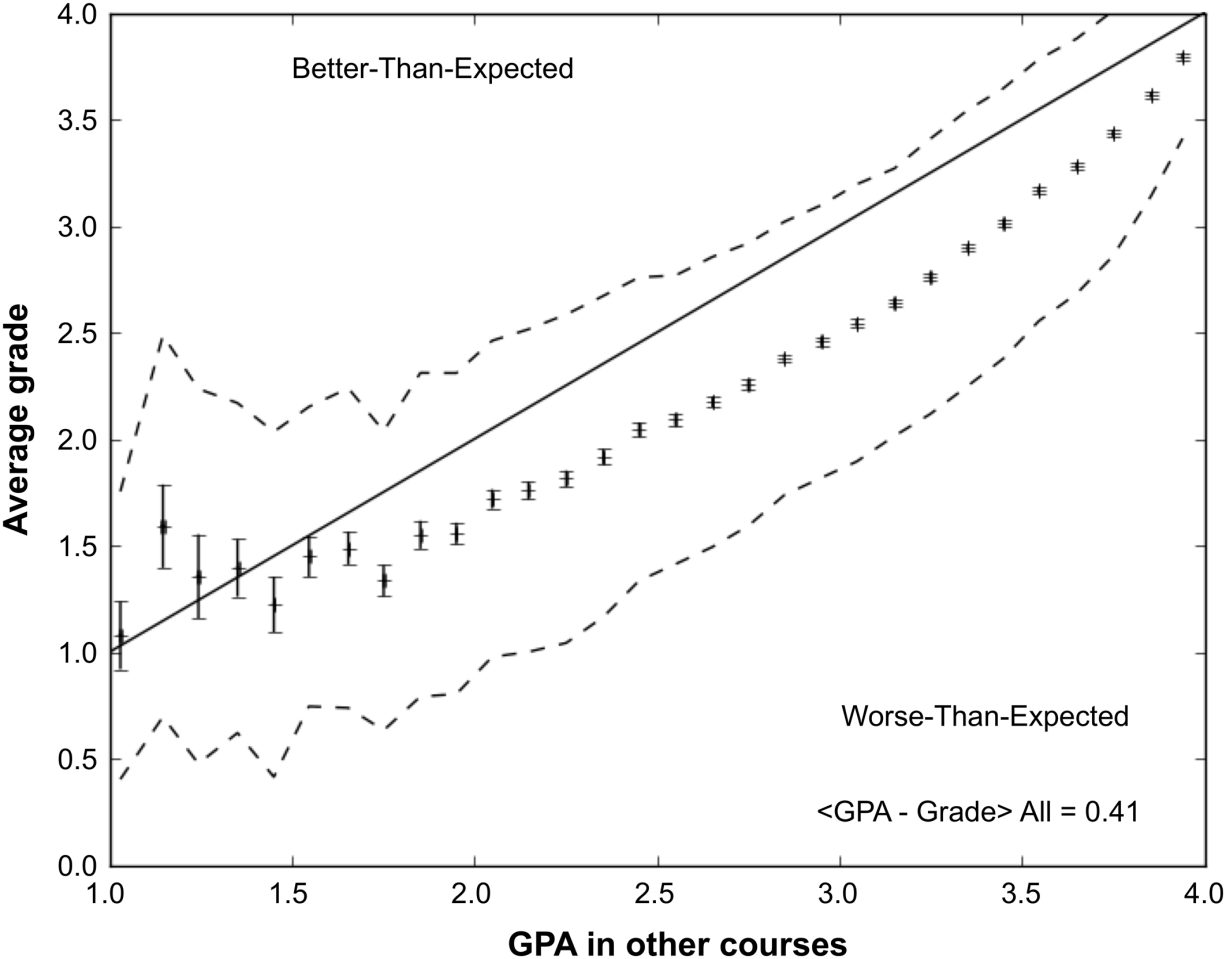
The relation between physics grade and GPA_other_ for students taking Physics 140. The points show mean grades for students in each bin of GPA_other_; error bars show the error on these means. The dashed lines show the one sigma dispersion in the relation between grade and GPA_other_. The grade penalty for this course is defined as the quantity GPA_other_—grade averaged over all students. For Physics 140, this grade penalty is 0.41 on a 4.0 scale.

For this reason, we adopt GPA as central to our approach to assessing student physics performance. At the end of the term, GPA is available for every student. We use this fact to generate a simple performance expectation for every student: the average actual performance of students with a similar GPA taking the course at the same time. An example of the relation between physics grade and GPA in other courses is shown in Fig 1. Students across the full range of GPA receive average physics grades lower than their grades in other courses. We have come to refer to this difference, averaged over all students, as the *grade penalty* of the course. On average, students who take physics will see their GPA decline. Grade penalties range from 0.2–0.6 letter grades in these courses. They are typically larger for female students: they enter all physics courses with higher GPAs and tend to underperform relative to their male peers [13].

## E^2^Coach and computer tailored communication

We created a tool, E^2^Coach, which aims to provide personalized support to all students in large introductory STEM courses. For the iteration of E^2^Coach discussed in this paper, the coaching focused on improving study techniques and habits, providing encouragement at appropriate times, and providing matched advice from other students. E^2^Coach did not address details of the course content. It was first introduced as an opt-in tool to four introductory physics classes in January 2012 and has been live in those classes for each subsequent full-length semester.

E^2^Coach communicates with students through personalized webpages, providing each with unique content based on the information we gathered about them. Information about the students comes to us in three primary ways. Basic student record information is gathered directly from the Office of the Registrar, while details of their current performance in physics are taken from the course grade book. Performance data—homework scores (using the Mastering Physics system), exam scores, and in-class performance scores (i>clicker scores)—are included to refine tailoring as the semester progresses. All remaining information is given to us by the students through surveys embedded in the E^2^Coach platform. The largest survey is the initial survey, completed by students when they opt-in to the system. It provides details about their background and preparation including their standardized test scores, advanced placement (AP) scores, present major at Michigan, and post-college plans. It also includes questions about their study habits, such as their planned office hours attendance, and their planned approaches for preparing for exams (e.g. number of hours, studying alone or with a partner) and regular studying (e.g. number of hours per week planned for studying). Also asked is what grade they want and how confident they are they can achieve it, and what their attitudes are about science in general, probed through adopted questions from the CLASS survey[14].

While all of this information is available, it is selectively and strategically used to create messages by a person, a *message author*. Historically, the *message authors* for this project have been recent graduates of the physics department (both undergraduate and graduate) who wrote the messages in consultation with a physics professor. Messages are written in a powerful, open-source software called the Michigan Tailoring System (http://chcr.umich.edu/mts/), developed by the Center for Health Communications Research at Michigan. Within the framework of this software, a *message author* can use tailoring logic to construct personalized messages for students. The end result typically consists mainly of text that reads similarly to the style of a personal letter, in which the text that a student sees depends on the data we have about them. Any amount of text (i.e. single words, sentences) may be sent to a student with a certain tailoring attribute, or direct substitutions may be made based on the information database (i.e. their name, or the number of hours they plan to study). For example, a physics 240 student who took AP physics in high school and has already joined study group at the Science Learning Center (SLC) may see the following text as a piece of his welcome message:

You have many strengths coming into this class. Some of the most important are:

- You’re signed up for a SLC study group—it’s great that you’ve already committed at least 2 hours every week to working in a group.
- You’re already in your second semester of physics in college—you understand what’s expected of you.
- You’ve already learned some Electricity and Magnetism in your high school AP class.

**Fig 2:**
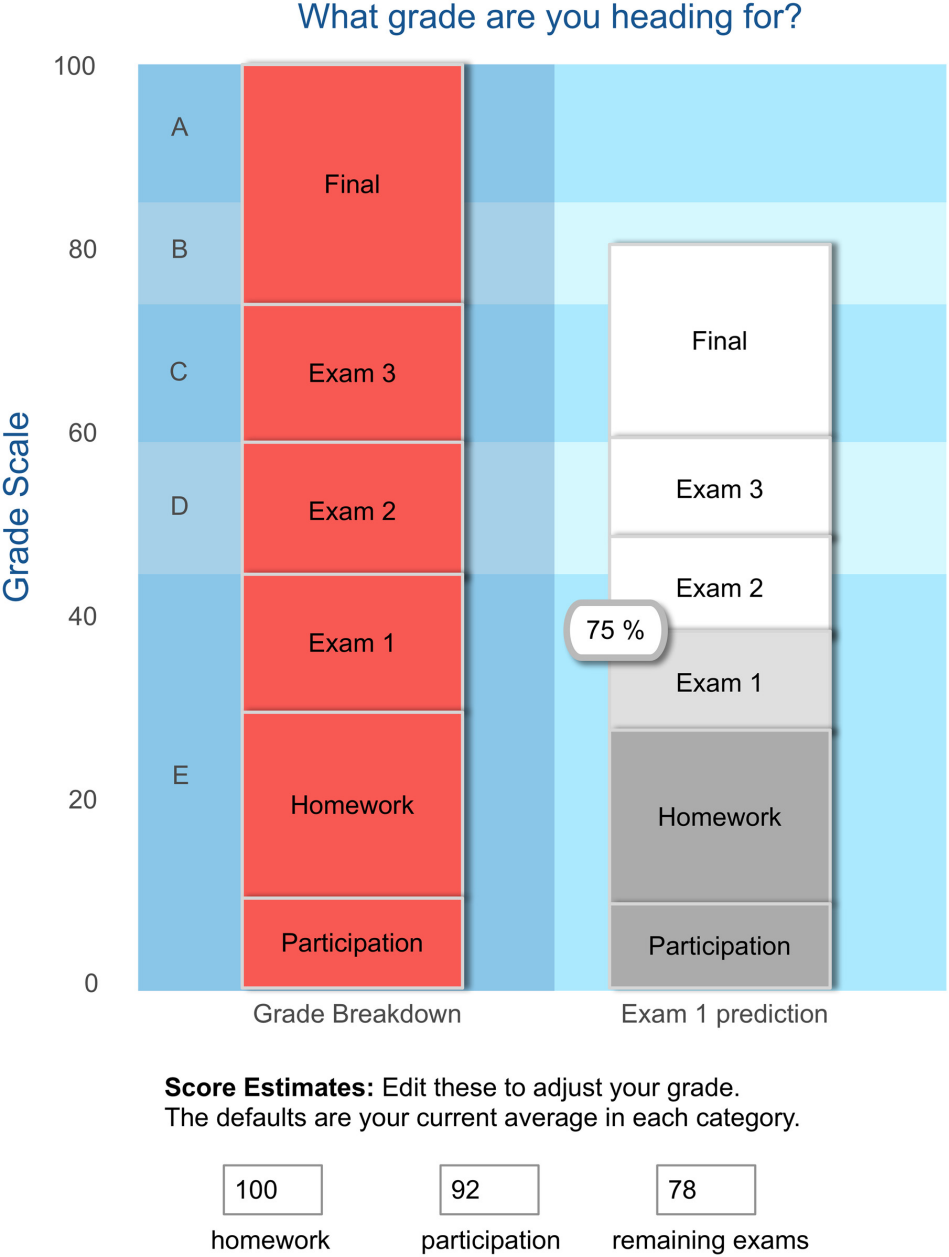
The grade prediction tool. The grade breakdown of the class is present on the left, and the height of the bar chart represents the predicted grade for the student. Roll-over features indicate the student’s specific scores in their exam, homework, or participation. In this case, the student received a 75% on exam 1. The initial prediction simply assumes the student will receive similar grades in the rest of the course.

**Fig 3:**
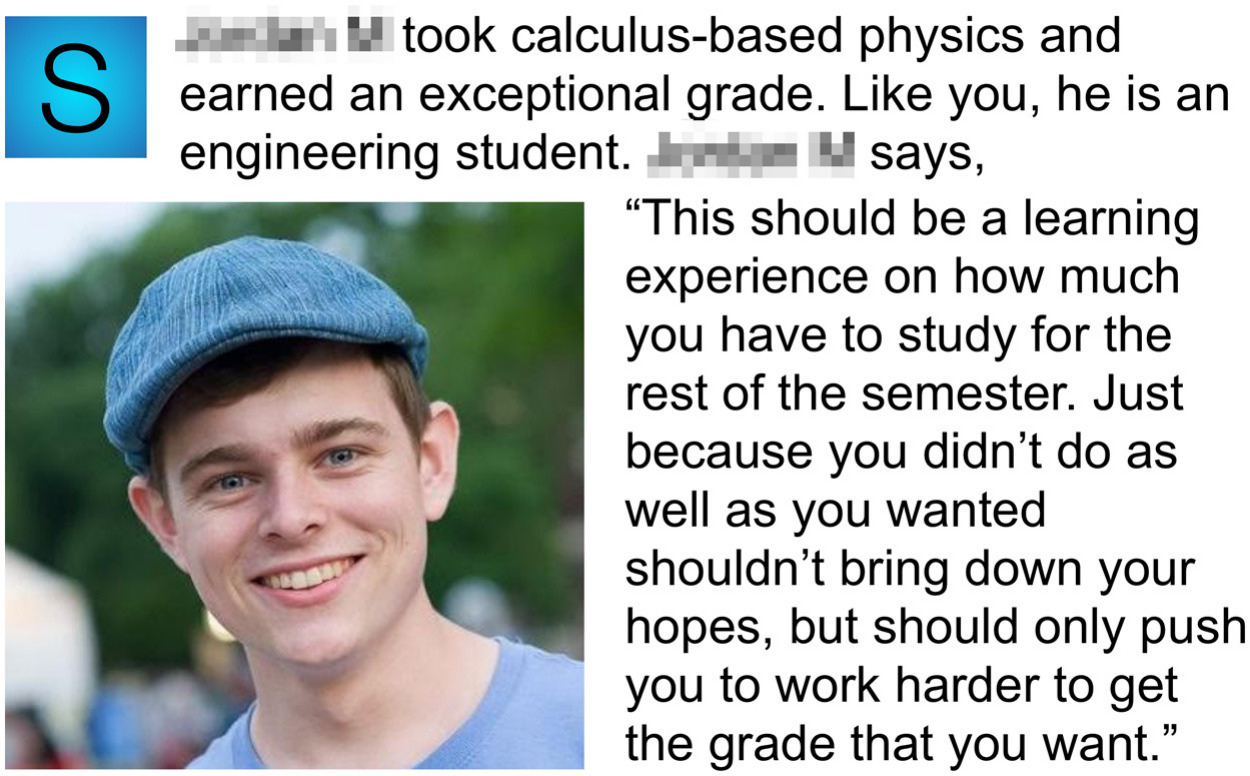
Peer advice sent to a student in response to their first exam. Consent for image publication from the pictured student was obtained.

While text through the voice of the *message author* is the primary source of tailoring, E^2^Coach also provides customized graphics displaying normative information about the study habits and recommendations of prior students, comparing each student’s planned study practices to those of other students who have received the grades they hope for. In addition, we provide a highly popular interactive grade prediction tool that shows each student their predicted grade based on their current performance and allows them to explore a range of possible outcomes should their future performance improve (Fig 2). And, as part of the Better-Than-Expected project [15], E^2^Coach contains a store of past student testimonials that are matched to current students based on gender, aspiration/major, and performance, providing them insight into the efficacy of changing study habits—true stories that illustrate how new approaches can lead to better-than-expected performance. A sample peer testimonial is shown in Fig 3.

New content was released to all student webpages at key moments during the semester: at the very beginning of the semester, a week before the first exam, immediately after the first, second, and third exams, and after the final. In our results and discussion, we describe comparisons of the performance of students who used E^2^Coach to varying degrees.

## Methods

### Participants

The first E^2^Coach system was launched in January 2012, providing support to students in four large introductory physics courses at Michigan. A slightly modified system was provided for the fall 2012 and winter 2013 semesters. This opt-in system was offered to all of the 5,523 students in these courses. A total of 2,234 students participated in the E^2^Coach system in their physics class, completing the incoming survey and engaging with E^2^Coach at a variety of levels.

### Ethics Statement

The University of Michigan IRB reviewed this project prior to its inception and declared it exempt on 9/13/2011, per the following federal exemption category:

EXEMPTION #2 of the 45 CFR 46.101. (b): Research involving the use of educational tests (cognitive, diagnostic, aptitude, achievement), survey procedures, interview procedures or observation of public behavior, unless: (i) information obtained is recorded in such a manner that human subjects can be identified, directly or through identifiers linked to the subjects; and (ii) any disclosure of the human subjects’ responses outside the research could reasonably place the subjects at risk of criminal or civil liability or be damaging to the subjects’ financial standing, employability, or reputation.

Students did not sign explicit consent, but the opt-in procedure informed them that their data would be used for study. GPA and course performance data about students who did not opt in to the study was used. Identifying information was collected and used in the intervention, but removed before analysis was conducted. Students were not given the option to opt out of this study entirely due to the IRB finding that this research is exempt under 45 CFR 46.101. (b). The University of Michigan IRB evaluated the opt-in/opt-out procedures used in this project.

### Usage Evaluation

Data on frequency of their visits to the E^2^Coach site and the length of their stay on each page, was collected and stored. To test the impact of E^2^Coach usage we divided the students into four groups: non-users who never signed up, along with low, medium, and high E^2^Coach users. Usage groups were based on the number of visits to E^2^Coach pages (above or below the median) and the number of unique weeks in which students visited. Low users were on the low side of both measures (with two or fewer weeks of visits), high users on the high side of both (with five or more weeks of visits), and moderate users filled the gap between the two.

### Data Collection

Much of the data that populated messages to E^2^Coach participants was pertinent information for our subsequent analysis, including students’ grades and GPAs from the Office of the Registrar, and details of their current performance in their class, which was continuously pulled from the course grade book to continually tailor messages for students over the course of the term. Data from student surveys administered in E^2^Coach was also available, which included their standardized test scores and AP exam scores prior to attending college. As a result, we have a relatively thorough academic portrait of students who enrolled in E^2^Coach and for all students of the courses, we collected performance data related to course grades and GPA from the registrar. The data from these terms, as well as author-generated metrics of achievement are available in the repository here: https://github.com/ecoach/plos_dataset.

### Better-Than-Expected Measure

Instead of relying solely on an absolute measure such as final grade to address student physics performance, E^2^Coach used a relative measure developed at Michigan: the “better-than-expected” score (BTE score). This score relies on our finding that incoming University of Michigan GPA is the strongest and most reliable predictor of student grades in physics. In addition, the psychology literature confirms that GPA is significantly and positively related to academic self-concept [16, 17]. Students think of their GPA when they identify what kind of student they are: “I’m a 3.5 student.” By adopting GPA as a predictor, we emulate the way in which a student might interpret their grade.

We construct our BTE score by comparing each student’s physics performance to that of other students in the class with a similar GPA_other_. A student who receives a grade higher than those of students with comparable GPA_other_ performs “better-than-expected”. Other students perform about “as expected” or “worse-than-expected” (WTE). Through the application of this measure, a student receiving a B- in physics might be considered “BTE” if her incoming GPA was 2.0, while a student with a 4.0 GPA receiving his first B+ would be considered “WTE”.

The BTE score is calculated by first measuring both the mean and the scatter in the grades in physics for students with each GPA_other_ in each semester, for each of the four classes, thus accounting for potential differences in grading patterns among the courses. Individual BTE scores are then derived according to the following relation, where $G_{exp}^{course}(GPA)$ is their predicted performance in the course:

$$\begin{array}{c} BTE\left(G_{actual},GPA\right)=\frac{G_{actual}-G_{exp}^{course}\left(GPA\right)}{\sigma_{exp}^{course}\left(GPA\right)} \end{array}$$ (1)

By design, the mean and standard deviation of BTE scores for all students used to form the relation $G_{exp}^{course}(GPA)$ should be zero and one: averaged across these courses they are measured to be 0.003 and 0.990. The distributions of all BTE scores are approximately normal, and very similar across semesters.

We used a quadratic fit for the regression model of BTE scores, which resulted in a slight reduction in the overall sum of residuals and a closer fit towards the center of the distribution when compared to linear fits, but at a cost of a slightly conservative grade estimate for users on the low and high end of the spectrum. Since it was harder for users on either end of the grade spectrum to achieve their grade prediction, they contributed slightly more often to negative BTE scores. The grading scale itself also imposes mobility constraints on students near the boundaries. However, both our quadratic prediction model and the unequal mobility of students on the grade scale are believed to be second order effects when compared to the primary effect, which is the average student’s motion in this BTE space. Hence, we primarily use the BTE measure as a relative measure of student performance.

## Results and Discussion

Descriptive characteristics of the sampled population of physics students are found in Table 4. Among E^2^Coach users in the low, moderate, and high usage groups, we found no significant differences in the levels of physics and math that students completed in high school, or in their SAT and ACT math scores. Hence, differences in students’ background physics and math levels do not seem to measurably inform students’ usage of E^2^Coach.

**Table 4:** Descriptive characteristics of the sampled population of physics students.

	**Total sample**	**Nonusers**	**Low users**	**Moderate users**	**High users**
Number of students	5523	3289	898	745	591
% Males	62.9	71.5	54.2	47.9	47.5
Average incoming GPA	3.01 (.99)	2.92 (1.05)	3.04 (.92)	3.15 (.87)	3.28 (.80)
Average SAT Math Score	-	-	725.52 (66.25)	724.07 (54.73)	730.60 (59.53)
Average ACT Math Score	-	-	31.39 (3.30)	31.79 (3.19)	31.56 (3.21)
**HS Physics Level**					
% No Physics	-	-	13.7	13.8	12.2
% Non-AP Physics	-	-	61.1	58.4	62.1
% AP Physics	-	-	25.2	27.8	25.7
**HS Math Level**					
% Non-AP	-	-	18.0	16.0	17.6
% AP Calculus AB	-	-	41.1	40.5	43.1
% AP Calculus BC or higher	-	-	39.2	42.1	36.5

Standard deviations are in parentheses. Statistics were calculated based on pairwise deletion of missing data. Inclusion in user groups required final completion of class. ACT math scores range from 1–36, while the SAT math score ranges from 200–800. High school physics level and math level data include the raw numbers of students in each of the groups.

Although GPA was significantly different across user groups, F(3, 5519) = 28.83, *p* <.001, the BTE score controlled for differences in GPA across user groups. Specifically, the BTE score compared the performance of each student in an E^2^Coach user group to a student of commensurate GPA in the nonuser group. Furthermore, all levels of GPA were present in all groups, so different levels of student performance were represented in each group.

### Effects of E^2^Coach Usage on Student Performance

We found no significant effects of physics course or semester in which the class was taken on mean BTE scores. Hence, we discuss the effects of E^2^Coach across these three semesters and physics courses combined.

**Fig 4:**
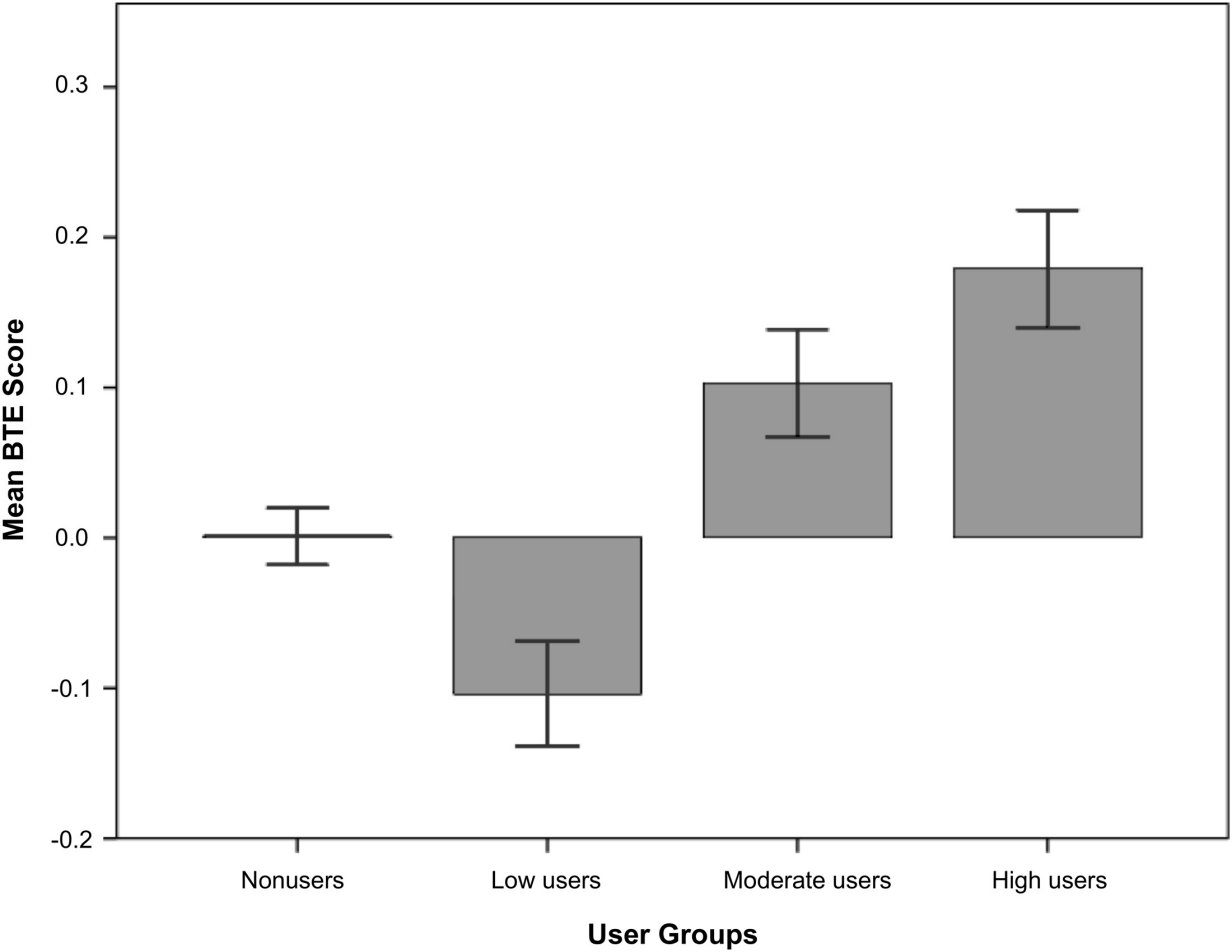
Mean BTE scores achieved across the four different user groups (nonusers, low users, moderate users, and high users). Error bars represent ± 1 standard error.

To evaluate the impact of E^2^Coach on student’s BTE scores, we ran a one-way analysis of variance (ANOVA). Mean BTE scores were significantly different across user groups, F(3, 4845) = 11.07, *p* <.001, as shown in Fig 4. Higher usage among users increased mean BTE scores in a linear fashion. This linear relationship was statistically significant, F(1, 4845) = 26.17, *p* <.001.

Which of these user groups had statistically different mean BTE scores? When taken together, users had higher BTE scores than nonusers, t(4845) = 20.4, *p* <.05. Within users, high users had higher BTE scores than low users, t(4845) = 5.22, *p* <.001. Moderate users also had higher BTE scores than low users t(4845) = 4.02, *p* <.001. High users did not have significantly higher BTE scores than moderate users, *p* = .17. Incidentally, we found that low users had lower BTE scores than nonusers,t(4845) = -2.65, *p* <.01. These planned contrasts were conducted with equal variances assumed, though the significance levels did not change with variances not assumed.

How do these gains in BTE scores translate into students’ physics letter grades? To compute this, we matched students within each user group with nonusers who had a GPA_other_ within +/- 0.1. Compared to non usage, high usage of E^2^Coach improved students’ letter grades by an average of 0.18 grades on the usual four-point GPA scale. Moderate users showed gains of .11 grades, and low users performed slightly below average at -.08 grade comparison. By matching students of commensurate GPAs for comparison, these gains in physics letter grades were independent of self-selection bias into the user groups.

Does usage have a significant effect on students’ BTE scores? Our results suggest that E^2^Coach usage was not only positively associated with student performance, but even accounted for students’ better-than-expected performance above and beyond their math and physics backgrounds. As proxies for students’ math and physics backgrounds, we used students’ ACT math scores, their highest level of math taken in high school, and their highest level of physics taken in high school, respectively. We conducted hierarchical multiple regression analyses: in the first step, HS physics and math level and ACT score were used to explain students’ BTE scores. In the second step, we added E^2^Coach usage, and found that it significantly improved the model (see Table 5). In fact, the effect of E^2^Coach usage on BTE scores was more than half as large as the effect of high school physics competency. Similar analyses were conducted with students’ final grade percentages—a more familiar measure of performance. E^2^Coach usage significantly accounted for 4.9% of the variance in students’ final percentage grades above and beyond indices of the high school physics and math competencies. Therefore, our results suggest that E^2^Coach usage can importantly contribute to student achievement in physics classes.

**Table 5:** E^2^Coach users have significant gains in BTE scores when controlling for high school physics level, high school math level, and ACT math score on BTE scores of E^2^Coach users (N = 1,412).

**Variable**	***B***	***β***	**Δ*R*^2^**
**Step 1**			.06**
ACT Math Score	.05**	.16	
HS Physics Level	.22**	.14	
HS Math Level	-.06	-.01	
**Step 2**			.01**
ACT Math Score	.05**	.15	
HS Physics Level	.21**	.14	
HS Math Level	-.01	-.01	
E^2^Coach Usage	.12**	.11	

** indicates statistical significance at the *p* <.01 level.

### E^2^Coach Usage and Gender

#### Gendered performance differences

Consistent with past literature on the gender gap in physics [18–20], there were gender differences in absolute physics grade, grade penalty, and BTE score in all of the introductory courses studied. Average grades, GPA_other_, grade penalty, and BTE score in each course for both male and female students are shown in Table 6.

**Table 6:** Average grades, GPA_other_, and BTE scores for students in the four physics courses studied.

Course	Grade	GPA_other_	GrPen	BTE	#	Group
135	2.80	3.19	0.39	0.00	7417	All
135	2.93	3.15	0.22	0.27	3118	Male
135	2.72	3.23	0.51	-0.19	4138	Female
235	2.91	3.25	0.34	0.00	5813	All
235	3.00	3.23	0.23	0.19	2501	Male
235	2.87	3.28	0.41	-0.12	3047	Female
140	2.71	3.11	0.40	0.00	11870	All
140	2.79	3.10	0.31	0.14	8284	Male
140	2.52	3.12	0.60	-0.33	3492	Female
240	2.75	3.12	0.36	0.00	9688	All
240	2.81	3.12	0.31	0.09	6948	Male
240	2.64	3.14	0.51	-0.24	2550	Female

Note: GrPen = ‘grade penalty’.

Of specific interest to the present paper, the gender difference in mean BTE score was significant, F(1, 4847) = 84.58, *p* <.001. A natural question that arises is whether these gender differences were driven by different degrees of high school physics and math proficiency. We, therefore, investigated such possible differences. See Table 7 for a summary of preparation by gender.

**Table 7:** Descriptive preparation characteristics by gender.

	Females	Males
SAT Math Score	712.32 (68.00)	736.25 (51.82)
ACT Math Score	32.63 (2.96)	33.86 (2.10)
**HS Physics Level**		
No Physics	196	102
Non-AP Physics	669	680
AP Physics	240	343
**HS Math Level**		
Non-AP	193	177
AP Calculus AB	465	460
AP Calculus BC or higher	418	462

As in Table 5, ACT math scores range from 1–36, while the SAT math score ranges from 200–800. Mean scores are presented with standard deviations in parentheses. High school physics level and math level data include the raw numbers of students in each of the groups. Table reflects data of E^2^Coach users.

We did not, however, find any interaction between gender and high school physics level, *p* = .80, nor between gender and high school math level on students’ BTE scores, *p* = .61. The more high school physics or math one had, the higher one’s BTE score in their college physics class. However, at every level of high school physics or math, males outperformed females on mean BTE score.

**Fig 5:**
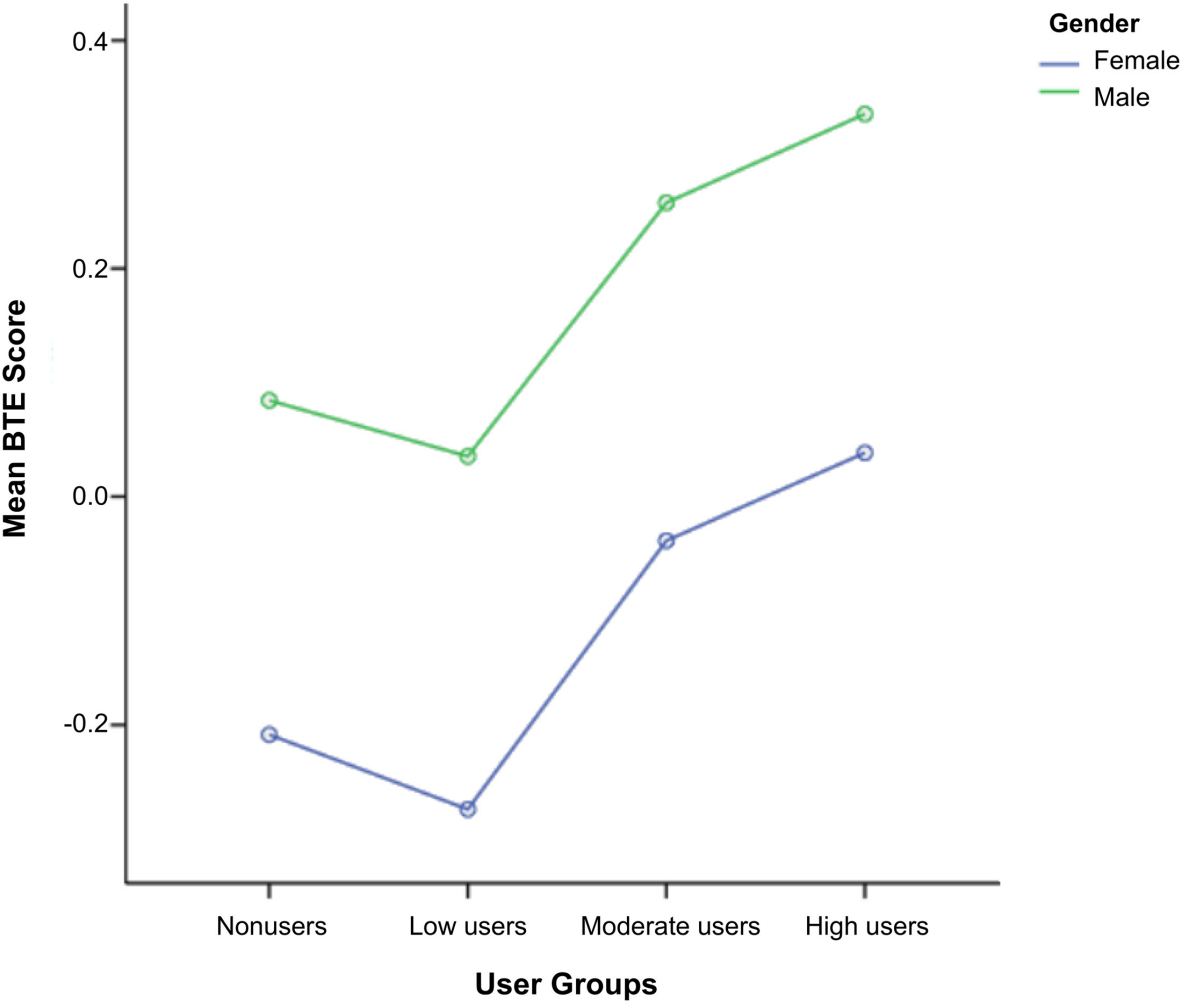
Students’ BTE performance as a function of gender and E^2^Coach usage across all user groups.

Given the gender differences in mean BTE scores, we tested whether E^2^Coach usage interacted with gender to affect students’ performance. AVONA showed that there was no significant interaction between gender and E^2^Coach usage, *p* = 1.00, but both main effects of gender and usage were statistically significant. Males had higher BTE scores, on average, across all user groups than females, F(1, 4841) = 76.25, *p* <.001. Higher E^2^Coach usage was positively associated with higher BTE scores for both males and females, F(1, 4841) = 16.66, *p* <.001. In other words, higher E^2^Coach usage increased mean BTE scores similarly for both males and females. These results are presented in Fig 5.

Despite our best intentions, E^2^Coach usage did not ameliorate the gender performance disparities seen in our historical data. Nonetheless, it did succeed in increasing both male and female students’ better-than-expected performance in their physics classes regardless of prior math and physics proficiency.

## Conclusion

In this paper, we argued for the importance of personalized support for students in large introductory physics classes. To meet this need, we created E^2^Coach, a computer tailored communication system which enabled us to give personalized feedback, encouragement, and advice to thousands of students. This system was used for three semesters in four large introductory physics courses at the University of Michigan. E^2^Coach aggregated information about each student’s background, goals, current status, and attitudes, and used it to construct messages unique to each student, delivered through personalized webpages. This system provided us with the opportunity to interact individually with students at scale.

To examine the impact of the system on student performance, we utilized a “better-than-expected” measure of performance that accounts for differences in student GPA, the most important predictor of performance. Since the E^2^Coach tool was offered to students as an opt-in system, usage varied substantially. For this reason, during analysis, we divided students into four usage groups and found that moderate and high usage of the system corresponded to higher BTE scores. We also explored the impact of the E^2^Coach system on performance and gender, and found that the relation between E^2^Coach usage and BTE scores was similar for male and female students.

It is important to note that the results reported here are correlational in nature. It is possible that students motivated to participate in E^2^Coach are also, independent of our coaching, more motivated to achieve success in physics. However, the use of BTE scores may ameliorate some effects of motivational confounds as the BTE score accounts for differences in GPA, differences which may reflect variations in motivation among different student groups. Indeed, high E^2^Coach users achieved improved performance when compared to nonusers even accounting for these differences in GPA. To more fully address the concern that motivation may be a confound, future versions of E^2^Coach should include fully randomized trials. Such trials are now being executed in an application of E^2^Coach to introductory statistics courses. The design of E^2^Coach naturally allows for randomized trials directly within the platform, in which, for example, students can be randomly assigned to conditions in which they receive more or less personalization in their messages. The effect of the degree of personalization on physics performance can both be broadly tested, with some students receiving a smaller degree of personalization, and others a larger degree, at every message, as well as tested for specific points of concern at distinct time points in the course. For example, the degree of personalization with regards to their study habits may be varied randomly for students at the E^2^Coach message sent out directly after the first exam, and examined against second exam performance along with analysis of behavioral data on their study habits administered as surveys after the first and second exams. Controlled trials such as these will be critical for further evaluation of the system.

One surprising result from our analysis was that low E^2^Coach users received significantly lower BTE scores than nonusers. The reasons for this particular finding can only be speculative due to limited data on how students actually changed their behaviors as the course progressed. Beyond the first detailed survey administered at the start of E^2^Coach no subsequent survey was required in two of the three semesters reported on here, so, even among high users though especially among low users, the amount of available data on student study behaviors over time is too low to enter into statistical tests. Future versions of E^2^Coach can require that students complete a survey on their study habits in order to see their personalized message. This would allow us to more closely monitor how reading the messages affects their study habits. Ideally as well, all students in the class, including nonusers, would fill out these surveys, so we can compare the behaviors of users and nonusers, as well as track each user over time. However, the cost of potential attenuation in E^2^Coach usage due to the desire to not fill out a survey should be monitored.

While these approaches can more clearly elucidate the effects of the E^2^Coach system, and personalized messaging, on student study habits, our findings from this first generation system provide a proof-of-concept for the use of such a system in the classroom. The version of the system we report on here was a first-generation system in which a relatively limited array of information was presented to students in a basic format. During the summer of 2013, we began a significant expansion in the use of E^2^Coach, enhancing the student interface in major ways and expanding its application to support students in physics, statistics, chemistry, and biology. While application to student support in individual classes like this is an important approach, we also look forward to the use of personalized communication like E^2^Coach in broader areas of student support, such as academic advising.
